# Instruction in Sex Hygiene—A Parent’s Problem*Read before the Public Health Educational Committee of the American Medical Association, at Yonkers, N. Y., February 17, 1913, and published in *The New York Medical Journal*, April 19, 1913.

**Published:** 1913-05

**Authors:** Mary Sutton Macy

**Affiliations:** New York


### Instruction in Sex Hygiene—A Parent's Problem.*

BY MARY SUTTON MACY, M. D., NEW YORK.

Fifty years ago, perhaps even twenty-five years ago, the parents who desired to instruct their growing children in subjects of sex

*Read before the Public Health Educational Committee of the American Medical Association, at Yonkers, N. Y., February 17, 1913, and published in *The New York Medical Journal*, April 19, 1913.

were so few as to be almost an unheard of phenomenon. Today the parents who wish so to guard their little ones are legion and they are incessantly putting to the doctors the questions, "What shall I tell?" "When shall I tell it?" and "How am I going to?" It is my purpose tonight to offer a few suggestions in answer to these questions, though I might summarize the answers thus: First—What shall I tell? The truth. Second—When shall I tell it? Always as soon as the child shows any curiosity or interest, perhaps first at two years of age, perhaps not till five or six, and from then on till maturity. Third—How am I going to? Simply, sincerely, earnestly and as a matter of course.

Let us consider these answers more carefully. You will say to me, "It is easy for you to say 'tell the truth,' but what is the truth and how much of the truth can I tell, or my child understand?" I am sorry that I have not time to go into all the details with you, but I can give you the names of some books which will instruct you in the detailed facts to be given, and I can give you a general statement of what truth must be told. The best of the books that I know are "The Mystery of Life" series, written by Dr. Isabelle Thompson Smart; but there is a whole host of books—good, bad and indifferent—which could be listed for you by any librarian, and from which you might get information and suggestions. I have limited my suggestion, because I know Dr. Smart's books are scientifically accurate and helpfully suggestive. The truth which you must tell consists in facts about the birth, the growth, and the functions of the whole body. Here and now let me emphasize the necessity of instructing children in the functions of the whole body, avoid overemphasis on sex, on digestion, on muscular activity, on circulation, or on the nervous or mental processes, and above everything avoid suggesting sex abnormalities, indigestion, laziness or overfatigue, heart disease, or morbid, nervous, or mental processes. More of this later; let me return to the truth you should tell. Every fact you give the child should be constructive, not destructive; hopeful, not discouraging; wholesome, not morbid; positive, not negative; and every fact you give must be adapted to the child's age, past mental experience, and present mental capacity. Common sense will tell any one that the amount of truth to be told to the two-year-old and to the fifteen-year-old is different; but I wish to emphasize that it is only the quantity and not the quality of truth which should differ.

Thus we have come to the second question, "When shall I tell it?" and the answer, "Always." To the baby's lisping question, "Where did baby come from?" do not lie. The fairy tales of stork or market basket, water spout, milk bottle, or ash can may be pretty symbols or humorous, teasing replies, but they are all harmful, and every one is liable to be found out sooner or later by the child and mercilessly judged in its true light as a lie, and once your child's confidence in your truthfulness is shaken, your control is lost and your power to influence the future life is forfeited.

The baby will be content usually with the answer, "From a seed, just as a flower comes from a seed," or "From an egg, as the birdie comes from an egg," according to the child's familiarity with flowers or with birds. The habit of "sleepy time tales" or "twilight talks" with your little ones has its value here, because long before the child has arrived at the age to ask, "Where does baby come from?" you can have taught facts about the flower's seed and the bird's egg. Nature stories may be deftly interspersed with real and favorite fairy tales which the children crave for, and nature stories can be begun in the old, old way, "Once upon a time." I am too devoted to Peter Pan and Snow White myself to desire to take the fariy tale out of the baby's life; all I ask is that you answer truthfully the child's earnest questions about life. The little one understands that a fairy tale is only make believe, but he cannot understand that mother or father is "making believe" when he asks earnestly for information and receives a false reply. The twilight talks have another good purpose if they are wisely guided; they serve to soothe the excited child, to relax the muscles, to quiet the fears, and to prepare the little body and mind to relax in peaceful sleep. To accomplish this the talks must not be ghostly, nor fearsome, nor in any way calculated to inspire terror or dread, and nature stories can serve in the desired manner, at the same time giving food for thought and laying foundations for future information of vital and definite kind. The habit of twilight talks established in babyhood is easily continued in later childhood years, and it is an easy and simple transition which makes it possible to incorporate in these hours, as the child grows older, tales and statements of facts which will answer questions asked or forming in the child's mind. Then, too, if during the day your little one asks for information you cannot give, because of ignorance or embarrassment difficult to overcome at the moment of asking, you can put off the question temporarily by the promise, "We'll take that up in our twilight hour tomorrow," or "day after tomorrow," according to what you may have already promised for the twilight hours, and according to the time you need to get the information necessary. Please let me sound a warning: Keep your word; do not put the child off with such a promise, and then forget or neglect to answer. Seek the information you need in the time you give yourself, or find out how to overcome your own embarrassment, as the case may be; but be prepared to answer questions when your child asks them, or promise at some definite time and then make good!

So we come to the third problem, "How to tell it." If you adopt the time of telling that I have suggested—that is, the twilight hour, or "sleepy time tale," your problem of how to tell things will be much simplified, since with the littlest ones the familiar "Once upon a time" will introduce to them any simple little tale, and it is easy to continue "there was a little boy" or "a little girl who wanted to know certain things." Clothing your information in simple language which the child can understand, leading the little mind along the path of knowledge from things which are known to it to the new, or as the scientific expression is, "from known to unknown," is the royal road to knowledge. One important thing to bear in mind in learning "how to tell" is not to tell too much. It is as dangerous to gorge the baby's mind with information as to gorge the little stomach with sweets, and the mental indigestion which is sure to follow "telling too much" is as hard to treat—yes, harder—as the physical indigestion from the sweets. Answer the little one's question truthfully, simply, but do not use the opportunity to dilate on allied topics, or to spread a feast of information before the eager mind. If you answer only the question asked, you will speedily be rewarded by more questions, until the curiosity is satisfied, and you will leave room for later experiences to develop a normal curiosity at a normal time in the future, and in a normal way. If you overanswer the question asked, you will stop questions for a time. That is, you will stop the child's questioning you, but start up a process of subjective, morbid questioning which will result in unwholesome mental habits and evil effects. If you underanswer the question asked you will be deluged with counter questions until you learn how to answer sufficiently in simple self-defense. If you fail or refuse to answer the question, some one will answer it for you, and probably in a way you would not desire. Therefore it is the part of wisdom to do yourself that which you would have well done. For the older child more detailed information is needed, and pictures, manikins, models, and such aids to instruction can be used to advantage. For this you will do well to prepare yourself beforehand by seeking personal instruction until you have mastered the practical details and knowledge, after which you will not find it hard to teach the child a little at a time when occasion serves.

All that I have said thus far applies to other truths than those of sex hygiene with equal power, but now I wish to say a few words of warning which apply most forcefully to instruction in sex. First and most important: Do not overemphasize it. Within the last decade and a half sex hygiene has come so prominently before the public mind—the adult mind—that we are in danger of making it too prominent to the child's mind. The pendulum has swung so far from the policy of telling the child nothing that we are in jeopardy of telling him too much. The child is entitled to sufficient information about the function of sex to save himself from the dangers and pitfalls of neglect and abuse, just as he is entitled to sufficient information on the subject of the digestive function to save himself from dyspepsia and other pitfalls of the kind, and sex and stomach should loom equally large in his horizon and no larger than muscle, or nerves, or mind in the abstract.

Secondly, in way of warning, avoid prohibitive "don'ts" in teaching the child of sex. To tell a little child, for example, not to masturbate is a most excellent way of leading him to experiment, for the purpose of finding out the reason for the "don't." Negative suggestion of this character is fruitful of much danger. Effective substitution of interest alone is the best and simplest way of arresting bad habits and of preventing their formation. Give the child other things to do with his hands, other games to play, other means of amusement than those involving the bad habit forming or formed. Constant care and watching will be necessary, if the habits are formed, and possibly change of environment to break up unwholesome companionships may be necessary; but avoid the "don't do that," and substitute the "do this" or "try this" in order to gain your end.

Third—In your instruction of the young child in sex matters avoid reference to the abnormal so long and so far as possible. If the child's personal observation results in questions concerning abnormalities, treat the immediate subject as you would a disease and let the child understand that such things exist as disease does, because of error in habit or in living, but do not be detailed in your information unless it cannot be avoided, and make your facts constructive in their trend. In all this instruction it is well to have as your motto:

"Look up, not down; look out, not in;
And lend a hand!"

Probably no living creature is more susceptible to suggestion than a little child, and it is frequently forgotten that the actions and objects of the child's environment, as well as the spoken "do's" and "don'ts" are as pregnant of possibilities for good and for evil. For this reason it is doubly important that the parents have a keen and constant oversight of the companions, caretakers, associates, and objects which surround their child. You cannot hope to keep from the little one all you would not desire him to know, but if you gain and retain his confidence you may so use even the undesirable but unpreventable facts and happenings of his environment that they may be positively useful and not detrimental in forming his character.

The price of wholesome minded, healthy children is good principles and eternal vigilance.

